# Tubercular Meningitis, Treated with Iodia

**Published:** 1878-07-20

**Authors:** J. W. Unger

**Affiliations:** Miss.


### SOUTHERN MEDICAL RECORD.

VOL. VIII. ATLANTA, GA., JULY 20, 1878. NUMBER 7.

EDITORS:

THOMAS S. POWELL, M. D. W. T. GOLDSMITH, M. D. R. C. WORD, M. D.

*R. C. WORD, M. D., Managing Editor.*

SUBSCRIPTION—$2.00 PER ANNUM, IN ADVANCE.

☞ All Communications and Letters on Business connected with THE RECORD, for 1877 and 1878, must be addressed to the Managing Editor.

### *ORIGINAL AND SELECTED.*

### TUBERCULAR MENINGETIS, TREATED WITH IODIA.

BY J. W. UNGER, M. D., OF MISS.

I was called to see Mr. H.'s child, age one year and four months, and found it in the following condition: Tongue coated with a whitish fur; slight frebrile movement; gums red and swollen; irritable and feverish. Without instituting a thorough examination, pronounced all due to difficult dentition, and thought there might be some malarial influence co-existing. I prescribed calomel as a purgative, with bromide potassium and sulphate of cinchonidia to allay nervous irritabillity, and to counteract any malarial impression, should any exist. Thinking this sufficient to entirely relieve the little patient of all trouble I left, and did not hear from it for several days, when I was again sent for and learned that it had been growing gradually worse. I protracted my stay in order to ascertain what the real trouble was. The mother was closely interrogated in the hope of eliciting information upon which to form a correct diagnosis—failing to procure any availiable or satisfactory information, I directed my attention to the child, and observed an alternate contraction and dilation of the pupil, with oscillation of the iris; this excited suspicion of miningitis. I placed my hand over the anterior fontanel to determine the amount of blood pressure, and found it full and forcibly pulsating. Knowing the intimate sympathy existing between the stomach and brain through the ganglionic system of nerves, I inquired whether the child had been observed to vomit at any time, and was informed it had occasionally, but it did not appear to suffer, before or after, any great nausea; which is characteristic of this sympathetic vomiting due to cerebral inflammation. I next inquired as to the conditlon of the bowels, and learned that during the initial stage, diarrhoæ existed, but subsequently constipation obtained.

Visit after visit revealed other very important features and manifestations, serving to corroborate and strengthen any conviction of the existence of miningitis. The *tache cerebrale* of M. Trousseau, on which he and others place so much reliance and value as a diagnostic sign, was present and well marked. This is a strange phenomenon, admitting of no easy explanation, but when seen is highly significant, and due consideration and prominence should be allowed it in the assemblage of multiform manifestations presenting in this insidious, though formidable malady. The hydrocephalic cry was occasionally heard, and, by way of parenthesis, let me here remark, that no one who ever hears it will allow memory to be so treacherous as to forget its distinguishing and characteristic peculiarity. The retraction of the abdomen, spoken of by Golis, was absent in my case. He claims through this sign, to be able to differentiate meningitis from essential fevers in its incipiency. Billiet and Barthis, think it rarely occurs, except in cerebral disease. The pulse at first was considerably accelerated, but subsequently became irregular in rythm and force; intermitting, about every eight or ten beats. When improvement began it fell below the normal standard. Respiration was disturbed by sighing and irregularity. There was a dry cough, but this was not constant. Thinking that a sufficient account bas been given, to establish the correctness of my diagnosis, I will now report the issue, with the treatment, which proved successful, and apologizes for its being sent for publication. From the commencement of the pyrexial period, to the close of the fever, occupied a period of over a month, when decided and equivocal evidence of improvement was manifested. I could not help thinking the apparent improvement was cheating me into a false security, but soon I had the satisfaction of seeing and knowing, that my hopes were not without foundation, and that convalescence was begun, if complete recovery was never attained. I entertained fears of a relapse, and so informed the parents, but ten or twelve months have elapsed since, and the child is living and seems to be in tolerable health. I attribute the causation to a scrofulous diathesis predisposing; excited by reflex irritation, from difficult dentition.

Treatment: In the first stage, I gave iodide and bromide of potassium freely and frequently for three or four days, which succeeded in calming the nervous irritabillity, and exercised a derivative influence on the brain. I gave calomel as a purgative. I also kept constantly applied a cold compress to the head. I had read of Battle & Co.'s Iodia, and thinking it an excellent combination, and containing the agent upon which nearly every practitioner in this disease relied, determined to test it in this case. I commenced its use the fourth or fifth day and persistently kept it up, and was rewarded with success through its use. While this was the main, other agents as auxiliaries were employed, when individual symptoms arose demanding their use. I know one case is insufficient to establish its value or efficiency in this disease, but by reporting this, others might be induced to try it, and thus determine its real value.

